# Supplementary material for: Semi-automated IT-scATAC-seq profiles cell-specific chromatin accessibility in differentiation and peripheral blood populations
Source: Nat Commun. 2025 Mar 17;16:2635. doi: 10.1038/s41467-025-57931-2 (PMC11914533; doi:10.1038/s41467-025-57931-2)
Supplement: Supplementary file 1 — Supplementary Information [file 41467_2025_57931_MOESM1_ESM.pdf]

Supplementary Table 1. Comparisons of scATAC-seq methods.

Methods	Tn5	Sequencing	Equipment Reliance	Indexing strategy	Error source	Manual labour	Throughput	Library preparation cost per cell ($)
**sci-ATAC-seq**	Custom-made, ≥96 Tn5	Custom-made adapters; Whole lane sequencing	Flow cytometry	Tn5 + indexed PCR	Sorting error + Barcode collision	Heavy	10^4	>1.00
**Plate-based scATAC**	Nextera	Nextera adapters; Whole lane sequencing	Flow cytometry	indexed PCR	Sorting error	Heavy	10^2-10^3	0.18
**Fluidigm C**	Nextera	Nextera adapters; Whole lane sequencing	Microfluidics	indexed PCR	Distribution error	Light	10^2	>1.00
**μATAC-seq**	Nextera	Nextera adapters; Whole lane sequencing	Takara iCELL8	indexed PCR	Barcode collision	Light	10^3	0.81
**10x Chromium**	Nextera	Nextera adapters; Mutiplex sequencing with other libraries	10x Chromium	indexed PCR	Barcode collision	Light	10^4	0.50-1.00
**Bio-Rad dsciATAC**	Custom-made, ≥96 Tn5	Custom-made adapters; Whole flow sequencing	Single-Cell Isolator	Tn5 + indexed	Barcode collision	Heavy	10^5	0.05-0.10
**EasySciATAC**	Custom-made, 384 Tn5	Mutiplex sequencing with other libraries	No	Tn5 + Ligation + indexed PCR	Barcode collision	Heavy	10^6	≤0.006
**IT-scATAC-seq**	Custom-made, 10-24 Tn5	TruSeq adapters; Mutiplex sequencing with other libraries	Flow cytometry	Tn5 + Two indexed PCR	Sorting error	Light	10^4-10^5	≤0.01

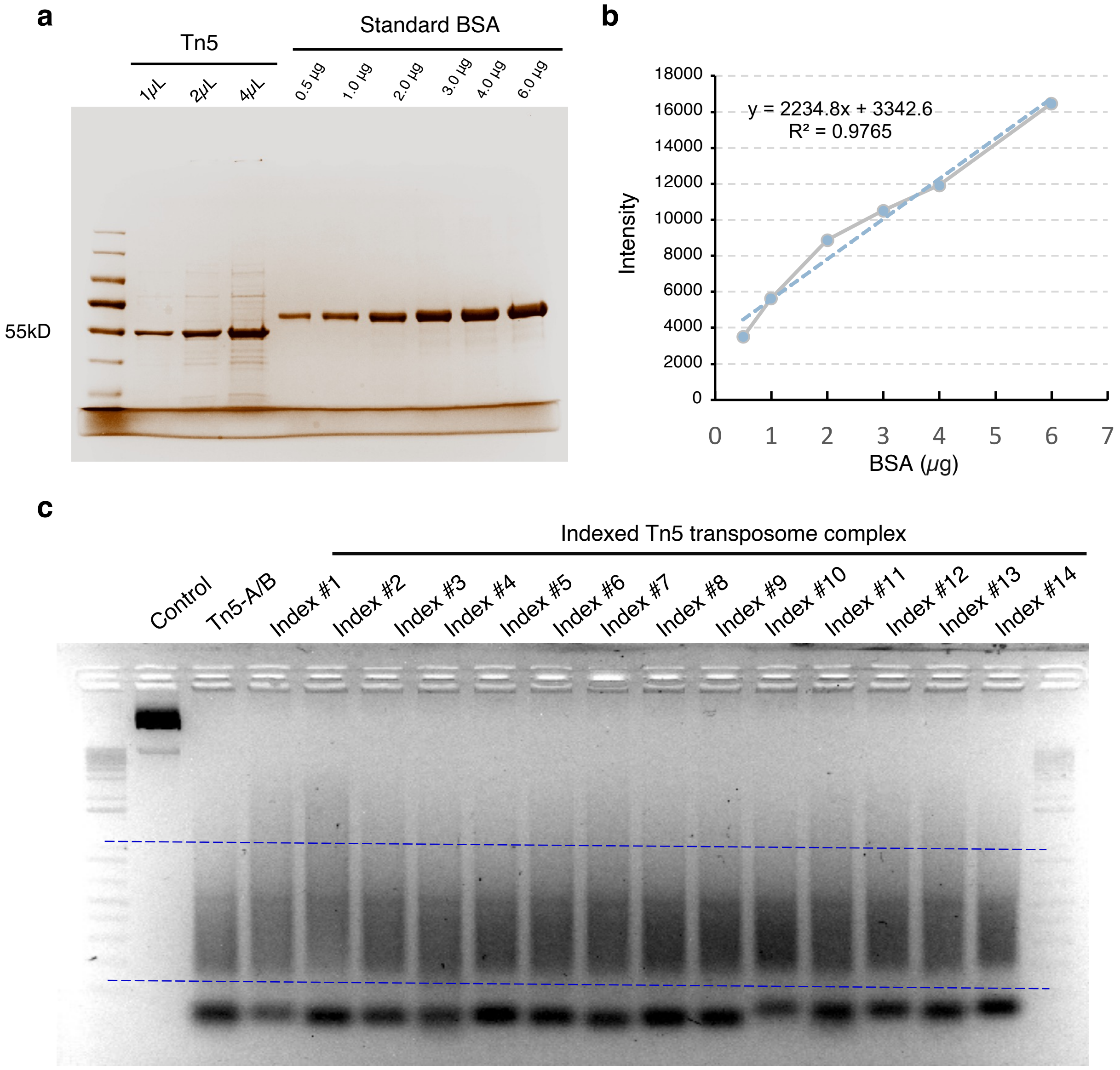

Supplementary Figure 1. Purification, assembly and quality control of indexed Tn5 transposome complex. a, SDS-PAGE and Coomassie blue staining of purified Tn5 (53.3 kDa), and a gradient of BSA were loaded for the standard curve plotting. **b,** The standard curve was fitted based on the loaded BSA, the equation, and the R-squared value calculation. **c,** Quality control of Tn5 activities by DNA electrophoresis of Tn5 transposome complex cleaved genomic DNA samples. 14 paired indexed Tn5 transposomes were randomly chosen for quality control, the undigested genome DNA as negative control and conventional A/B assembled Tn5 (Tn5-A/B) as positive control; the up and down bluelines correspond to 1kb and 100bp size. Source data are provided as a Source Data file.

a The 1st Indexing

Mix Tn5: Q5XX:Q7XX=2:1:1 to form indexed Tn5 transposome complex

Q5XX ***Tn5 Binding adapter***

5'-GGCGGTAGGCGTGNNNNNNNNAGATGTGTATAAGAGACAG-3'

3'-TCTACACATATTCTCTGTC-5'

Q7XX ***Tn5 Binding adapter***

5'-CCAACACCCGTGCGNNNNNNNNAGATGTGTATAAGAGACAG-3'

3'-TCTACACATATTCTCTGTC-5'

b The 2nd Indexing

H5XX

ACACTCTTTCCCTACACGACGCTCTTCCGATCTNNNNNNNNGGCGGTAGGCGTG

H7XX

GTGACTGGAGTTCAGACGTGTGCTCTTCCGATCTNNNNNNNNCCAACACCCGTGCG

c The 3rd Indexing

T5XX

AATGATACGGCGACCACCGAGATCTACACNNNNNNNNACACTCTTTCCCTACACGACGC

T7XX

CAAGCAGAAGACGGCATACGAGATNNNNNNNNGTGACTGGAGTTCAGACGTGTGC

d

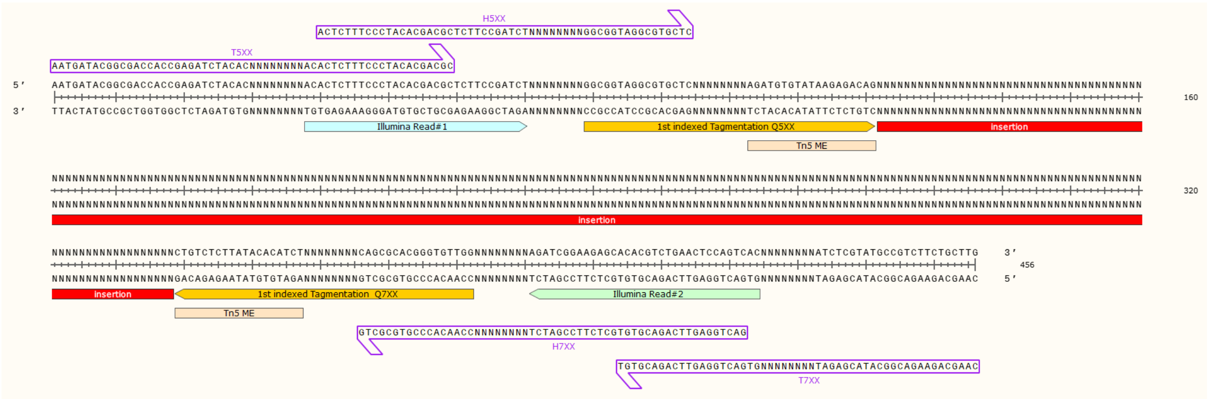

Supplementary Figure 2. Sequences of IT-scATAC-seq adapters and library structure. a, Indexed Tn5 transposome complex associated adapters, including indexed left adapters Q5XX and indexed right adapters Q7XX. After assembly Tn5 with unique Q5XX or Q7XX, mix equal Tn5-Q5XX and Tn5-Q7XX to form a paired Tn5-Q5XX/Q7XX. The first round of cell indexing was introduced by different paired Tn5 tagmentation. **b,** Sequences of the first PCR primers H5XX/H7XX targeting to Q5XX and Q7XX, respectively. A different combination of H5XX/H7XX for the second round of cell indexing. **c,** Sequences of the third cell indexing primers T5XX/T7XX targeting to H5XX/H7XX, respectively. The third round of cell indexing was introduced by a different combination of T5XX/T7XX. **d,** IT-scATAC-seq integrates the Illumina TruSeq Read 1 and Read 2 adapters in the library structure ready for the standard massively parallel sequencing, which overcomes the inherent inconvenience of custom sequencing requirements and reduces sequencing costs.

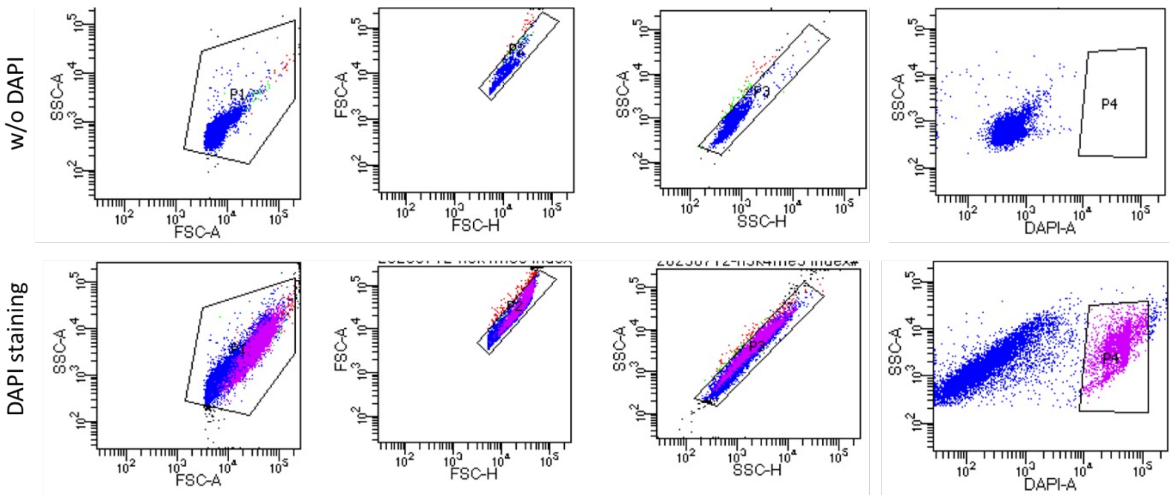

Supplementary Figure 3. Gating strategy for flow cytometry of DAPI stained nuclei.

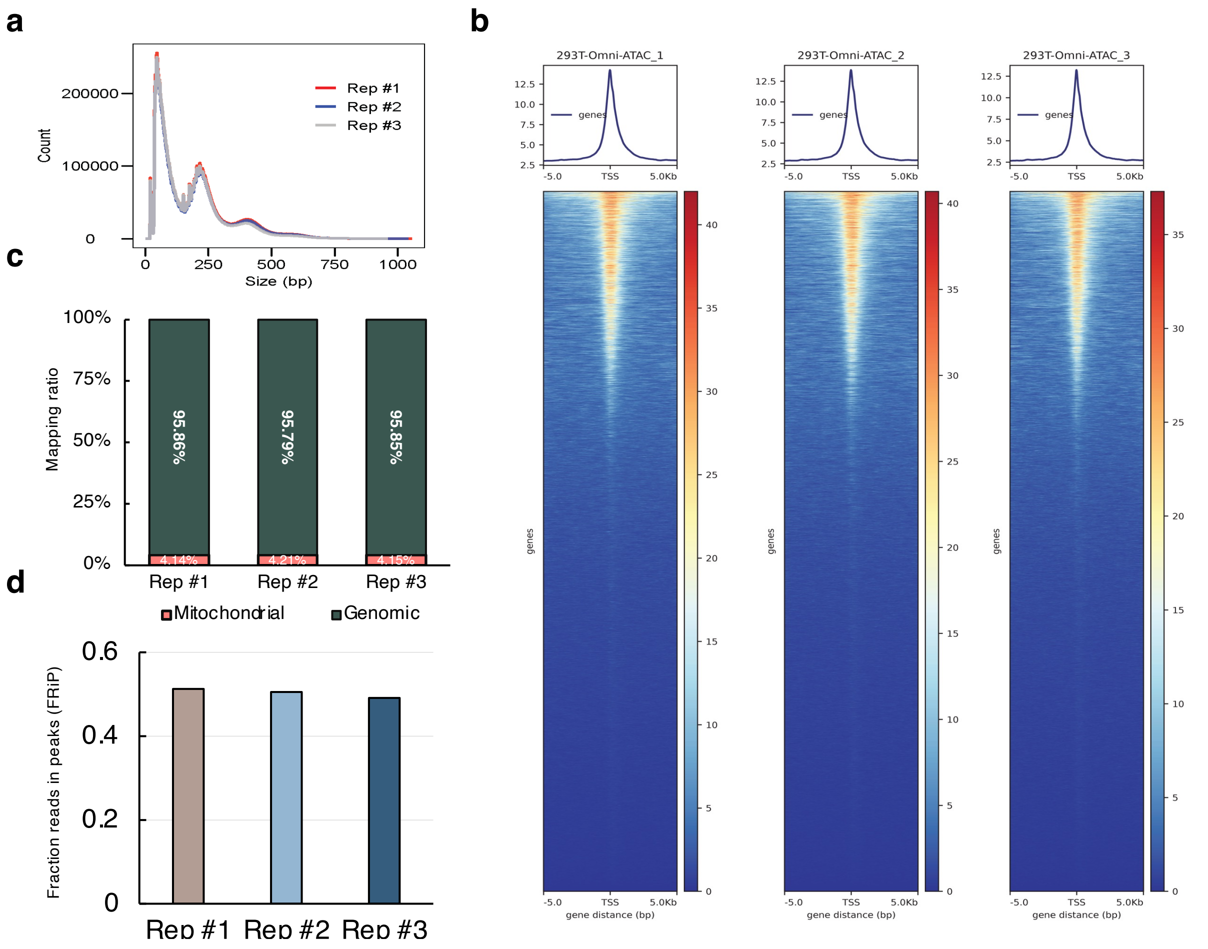

Supplementary Figure 4. Quality control analysis of bulk OmniATAC-seq of HEK293T(n = 3 independent experiments). a, Bulk HEK293T OmniATAC-seq fragment size distribution showing typical nucleosome periodicity pattern. **b,** Average signal and heatmap centred on the transcription start sites(TSS)showing ATAC-seq signals for bulk OmniATAC repeats. **c,** Mapping rate of bulk OmniATAC repeats to genomic and mitochondrial regions. **d,** FRiP of bulk OmniATAC repeats.

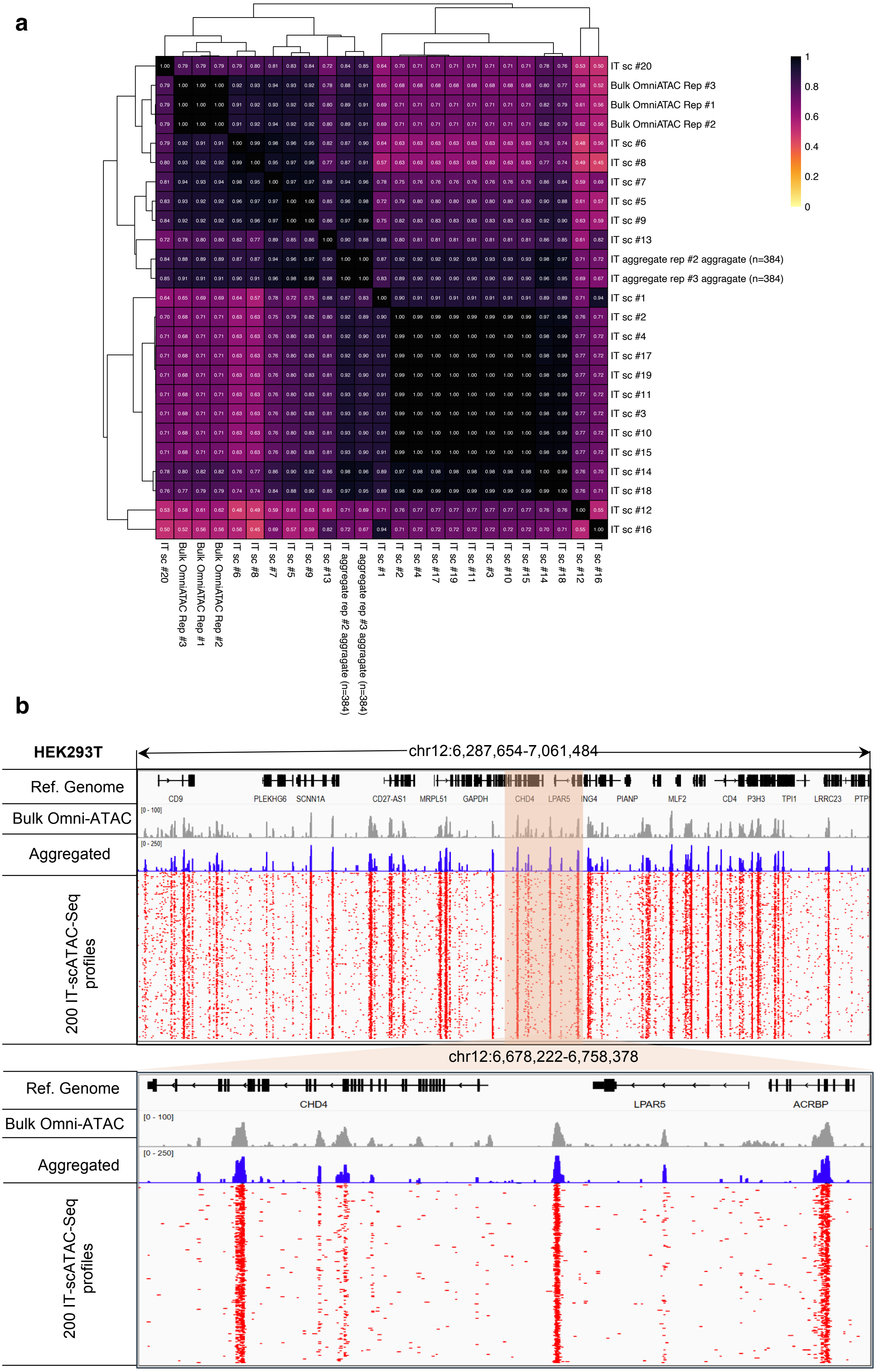
a
IT sc #20
Bulk OmniATAC Rep #3
Bulk OmniATAC Rep #1
Bulk OmniATAC Rep #2
IT sc #6
IT sc #8
IT sc #7
IT sc #5
IT sc #9
IT sc #13
IT aggregate rep #2 aggragate (n=384)
IT aggregate rep #3 aggragate (n=384)
IT sc #1
IT sc #2
IT sc #4
IT sc #17
IT sc #19
IT sc #11
IT sc #3
IT sc #10
IT sc #15
IT sc #14
IT sc #18
IT sc #12
IT sc #16
1
0.8
0.6
0.4
0.2
0
b
HEK293T
chr12:6,287,654-7,061,484
Ref. Genome
Bulk Omni-ATAC
Aggregated
200 IT-scATAC-Seq profiles
CD9
PLEKHG6
SCNN1A
CD27-AS1
MRPL51
GAPDH
CHD4
LPAR5
ING4
PIANP
MLF2
CD4
P3H3
TPI1
LRRC23
PTP
[0 - 100]
[0 - 250]
chr12:6,678,222-6,758,378
Ref. Genome
Bulk Omni-ATAC
Aggregated
200 IT-scATAC-Seq profiles
CHD4
LPAR5
ACRBP
[0 - 100]
[0 - 250]

Supplementary Figure 5. Assessment of IT-scATAC-seq libraries against bulk OmniATAC-seq. a, Heatmap showing Pearson correlation between bulk HEK293T OmniATAC-seq triplicates(Bulk OmniATAC-seq Rep #1-3, 20 single-cell IT-scATAC-seq profiles(IT sc#1-#20), IT-scATAC-seq replicates single-cell aggregates (IT aggregates rep#1 and rep #2). **b,** Genome tracks around CHD4 gene locus, showing aggregate and representative single-cell ATAC-seq signals (n = 200).

a

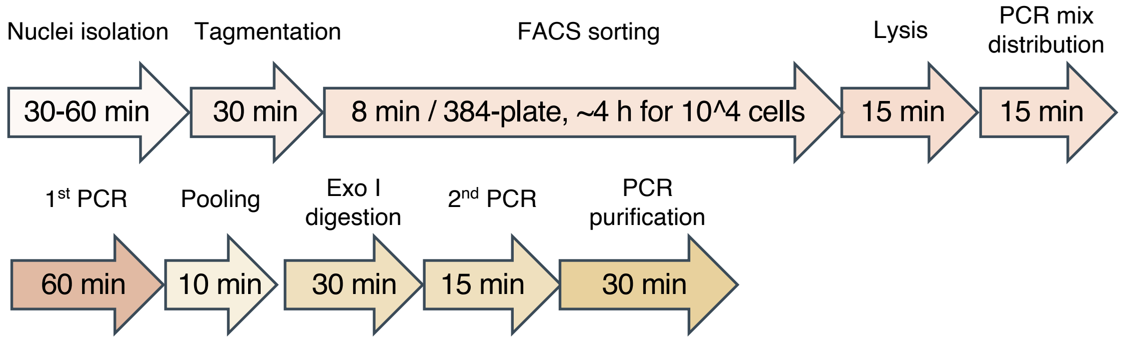

b

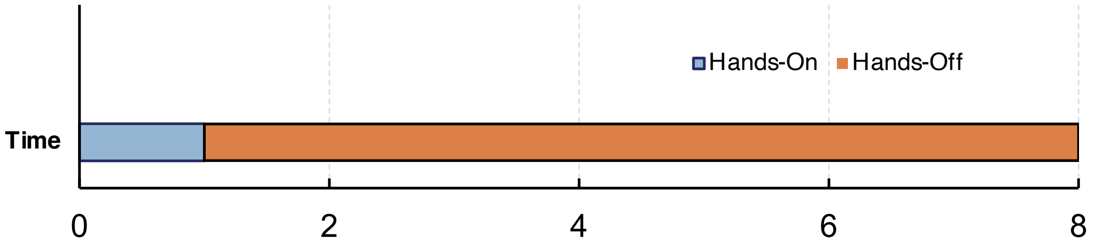

c

**Library preparation for 10^4 cells**	
Material	Cost / HKD
Tn5	20-100
384-plate	25-50
Oligos	20
2x NEB Q5 Mastermix	120
Flow cytometry*	280
Echo system	15
Exo I	5
Others	35
Total	< 600 HKD ($0.01/cell)

Supplementary Figure 6. Timeline and cost of IT-scATAC-seq. a, The timeline of IT-scATAC- seq library preparation for 10^4 cells. **b,** The fraction of time spent on hands-on and hands-off library preparation processes. **c,** Estimated total cost for IT-scATAC-seq library preparation covering 10^4 cells. *The cost of FANS is calculated by the equipment use charge per hour, which varies between institutes and labs.

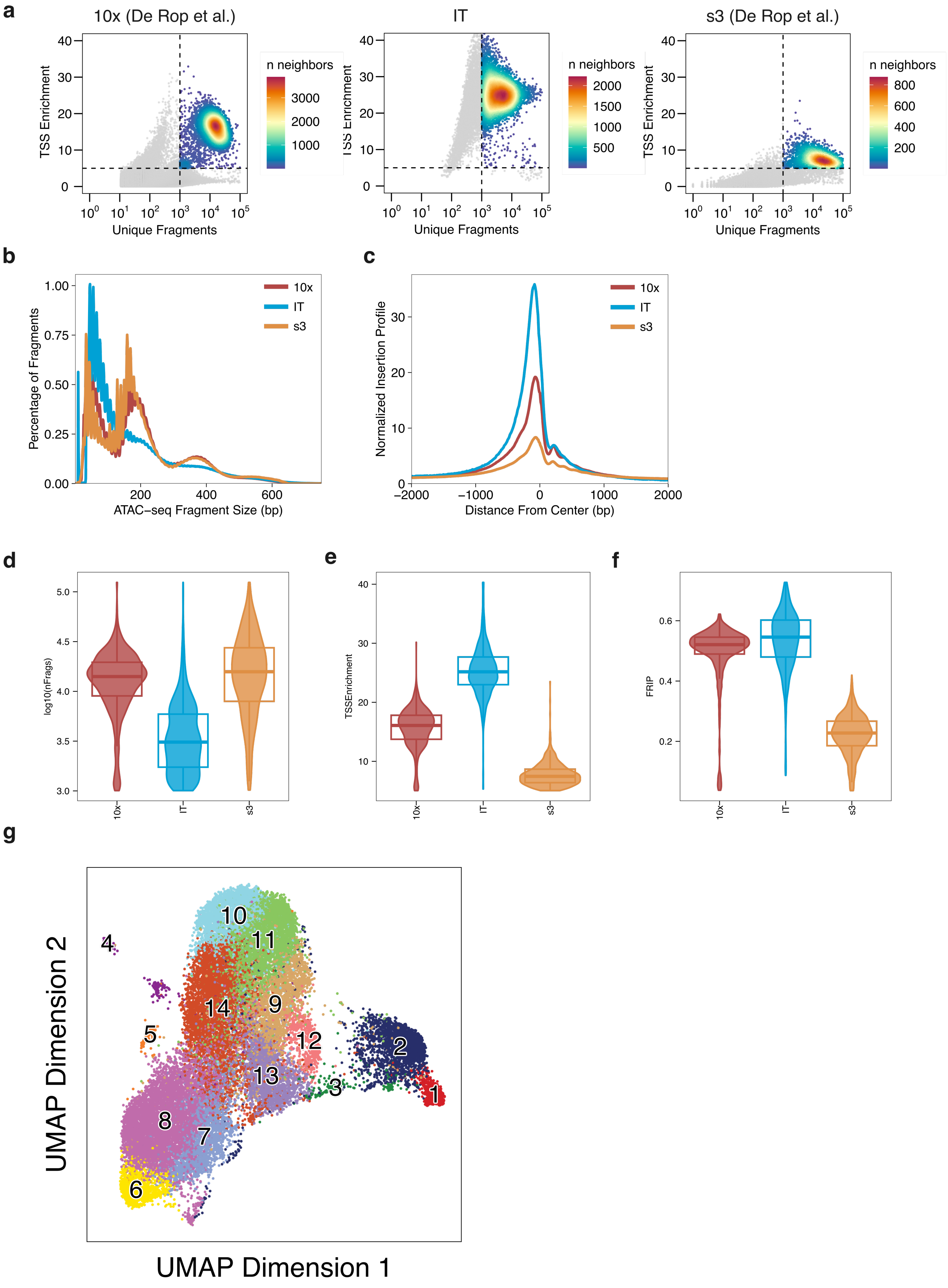
a
10x (De Rop et al.)
IT
s3 (De Rop et al.)
TSS Enrichment
Unique Fragments
n neighbors
b
10x
IT
s3
Percentage of Fragments
ATAC−seq Fragment Size (bp)
c
Normalized Insertion Profile
Distance From Center (bp)
d
log10(nFrags)
e
TSSEnrichment
f
FRIP
g
UMAP Dimension 2
UMAP Dimension 1

Supplementary Figure 7. Quality control and dimension reduction of PBMC scATAC-seq datasets profiled by IT-scATAC-seq (IT, n = 7,628 single cells), 10X Chromium(10X, n = 9,411 single cells), and s3-ATAC-seq (s3, n = 2,855 single-cells). a, TSS enrichment score plotted by the number of unique nuclear fragments for each sample. **b,** Nucleosomal periodicity is shown by the fragment size distribution of aggregate single-cell profiles of scATAC-seq libraries. **c,** Enrichment of normalised Tn5 insertions around the TSSs of the aggregate scATAC single-cell profiles. **d-f,** Violin plots displaying the distribution of log10-transformed unique ATAC-seq fragments per single cell (d), single-cell TSS enrichment scores (e), and Fraction of Reads in Peaks (FRiP) (f). In each violin, the central line represents the median, and the box bounds indicate the interquartile range (IQR, 25th–75th percentile). **g,** UMAP plots showing scATAC-seq profiles of three human PBMC data coloured by unannotated cluster after dimension reduction and batch correction.

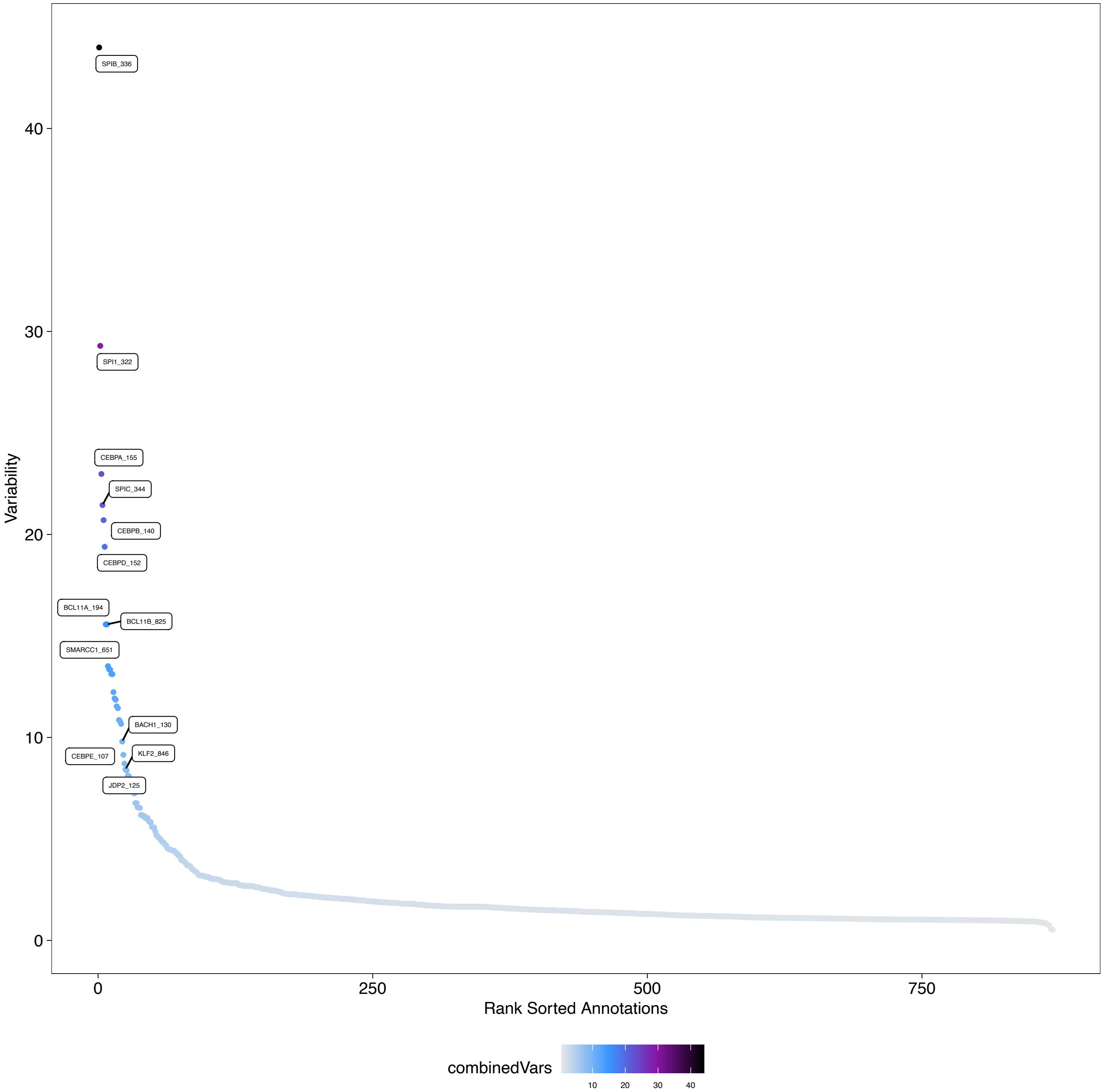
SPIB_336
SPI1_322
CEBPA_155
SPIC_344
CEBPB_140
CEBPD_152
BCL11A_194
BCL11B_825
SMARCC1_651
BACH1_130
CEBPE_107
KLF2_846
JDP2_125
40
30
20
10
0
Variability
0
250
500
750
Rank Sorted Annotations
combinedVars
10
20
30
40

Supplementary Figure 8. PBMCs showed diversified motif enrichment across subtypes of cells. Highly variable motifs associated with chromatin accessibility identified based on the per-cell motif activity score calculated by the chromVAR algorithm across all PBMC cell types.
