# Supplementary material for: Semi-automated IT-scATAC-seq profiles cell-specific chromatin accessibility in differentiation and peripheral blood populations
Source: Nat Commun. 2025 Mar 17;16:2635. doi: 10.1038/s41467-025-57931-2 (PMC11914533; doi:10.1038/s41467-025-57931-2)
Supplement: Supplementary file 2 — Description of Additional Supplementary Files [file 41467_2025_57931_MOESM2_ESM.pdf]

Description of Additional Supplementary Files

Supplementary Data 1. Sequences of IT-scATAC-seq adapters and library structure.

Supplementary Data 2. PBMC celltype specific differential accessible regions (DARs). Peakset called using MACS2 and DARs (FDR ≤ 0.1 & $\log_2$ fold change >1) across lineages were found under with TSS enrichment and the number of unique fragments per cell adjusted under Wilcoxon Rank-Sum Test.

Supplementary Data 3. Motif activity score calculated by the chromVAR.
