# Supplementary material for: Semi-automated IT-scATAC-seq profiles cell-specific chromatin accessibility in differentiation and peripheral blood populations
Source: Nat Commun. 2025 Mar 17;16:2635. doi: 10.1038/s41467-025-57931-2 (PMC11914533; doi:10.1038/s41467-025-57931-2)
Supplement: Supplementary file 6 — Reporting Summary [file 41467_2025_57931_MOESM6_ESM.pdf]

nature portfolio

Corresponding author(s): Jin Wei, Zhou Zhongjun

Last updated by author(s): Mar 5, 2025

Reporting Summary

Nature Portfolio wishes to improve the reproducibility of the work that we publish. This form provides structure for consistency and transparency in reporting. For further information on Nature Portfolio policies, see our Editorial Policies and the Editorial Policy Checklist.

Statistics

For all statistical analyses, confirm that the following items are present in the figure legend, table legend, main text, or Methods section.

n/a	Confirmed	
☐	☒	The exact sample size (*n*) for each experimental group/condition, given as a discrete number and unit of measurement
☐	☒	A statement on whether measurements were taken from distinct samples or whether the same sample was measured repeatedly
☐	☒	The statistical test(s) used AND whether they are one- or two-sided<br>*Only common tests should be described solely by name; describe more complex techniques in the Methods section.*
☐	☒	A description of all covariates tested
☐	☒	A description of any assumptions or corrections, such as tests of normality and adjustment for multiple comparisons
☐	☒	A full description of the statistical parameters including central tendency (e.g. means) or other basic estimates (e.g. regression coefficient) AND variation (e.g. standard deviation) or associated estimates of uncertainty (e.g. confidence intervals)
☐	☒	For null hypothesis testing, the test statistic (e.g. *F*, *t*, *r*) with confidence intervals, effect sizes, degrees of freedom and *P* value noted<br>*Give P values as exact values whenever suitable.*
☒	☐	For Bayesian analysis, information on the choice of priors and Markov chain Monte Carlo settings
☐	☒	For hierarchical and complex designs, identification of the appropriate level for tests and full reporting of outcomes
☐	☒	Estimates of effect sizes (e.g. Cohen's *d*, Pearson's *r*), indicating how they were calculated

Our web collection on statistics for biologists contains articles on many of the points above.

Software and code

Policy information about availability of computer code

Data collection	Bio-Rad image lab ChemiDoc XRS+ system for western blot. BD FACS Aria-TM for flow cytometry sorting. ATAC-Seq datasets were collected by Illumina NovaSeq.
Data analysis	Single-cell ATAC-seq data pre-processing. Cutadapt64 4.5 was used to remove TruSeq Index 1 (i7) Adapters and Index 2 (i5) Adapters at both 5'- and 3'-end of each read. The barcode sequences were then extracted from 5'-end of each read sequence and appended to read headers of the paired-end reads by Cutadapt 4.5 with --rename=CB:Z: (r1.adapter_name)(r2.adapter_name) -e 0.04 --no-indels --action=trim, and adapter sequences and name are specified in FASTA files with parameters -g and -G. The trimmed and barcode-extracted reads were mapped to the corresponding reference genomes, including human (GRch38) for HEK293T and human PBMCs, and human (GRCh38) and mouse (mm10) hybrid genome assembly for species-mixing experiments, using BWA-MEM65 v.0.7.17. The bam file is then sorted by the cell barcode (CB) tag and split into BAM file by CB using SAMtools66 1.17. MarkDuplicates of Picard Tools 3.1.0 was used to mark and remove duplicated reads for the demultiplexed BAM file for each single cell. The deduplicated BAM were then merged using SAMtools into a deduplicated single-cell aggregate BAM file for downstream analysis. Using deduplicated single cell aggregates BAM file, accessible chromatin regions (peaks) were called using MACS267, with parameters -f BAMPE -g hs --shift -75 --extsize 150 --nomodel --call-summits --nolambda --keep-dup all -p 0.01 -B.<br>Bamcoverage of Deeptools suite (version 3.5.2) was used first to normalise total reads to 10,000,00 and generate BigWig and Bedgraph files with the parameters --scaleFactor 10,000,000/reads_number --binSize 50. We used Deeptool's multiBigwigSummary and plotCorrelation to calculate the Pearson correlation coefficient between the normalised single-cell aggregate, randomly selected single-cell profiles and bulk omniATAC-seq of HEK293T.<br>Comparison with existing scATAC-seq methods performing on cell lines. Quality control metrics were obtained from plate-based methods to compare quality control metrics with the plate-based and C1-based methods8(https://github.com/dbrg77/plate_scATAC-seq). Fragments files were downloaded from the Sequence Read Archive (SRA) or Github repository and imported to ArchR to calculate the quality control metrics

for sci69(GSE109828), CH-based ATAC-seq33(https://bis.zju.edu.cn/chatac/), HydropATAC32(GSE175684). 10X GM12878 and A20 Cells quality control metrics were obtained from www.10xgenomics.com/datasets/.

For manuscripts utilizing custom algorithms or software that are central to the research but not yet described in published literature, software must be made available to editors and reviewers. We strongly encourage code deposition in a community repository (e.g. GitHub). See the Nature Portfolio guidelines for submitting code & software for further information.

Data

Policy information about availability of data

All manuscripts must include a data availability statement. This statement should provide the following information, where applicable:

- Accession codes, unique identifiers, or web links for publicly available datasets
- A description of any restrictions on data availability
- For clinical datasets or third party data, please ensure that the statement adheres to our policy

The datasets supporting the conclusions of this article are available in the NCBI Sequence Read Archive (https://www.ncbi.nlm.nih.gov/sra) with accession number PRJNA1073020. The accession number accession number for each dataset is SRR32538998 (HEK293T cell line), SRR32538997 (K562 cell line), SRR32538996 (H1 cell line), SRR27862248 (mixed species using HEK293T and NIH/3T3), SRR28081828 (mouse ESCs differentiation), and SRR28081827 (human PBMCs).

Research involving human participants, their data, or biological material

Policy information about studies with human participants or human data. See also policy information about sex, gender (identity/presentation), and sexual orientation and race, ethnicity and racism.

Reporting on sex and gender	N/A
Reporting on race, ethnicity, or other socially relevant groupings	N/A
Population characteristics	N/A
Recruitment	Two healthy donors were recruited for an annual physical examination.
Ethics oversight	The donors signed with the informed consent given by guardians and human tissue procurement under the guidance of ethical regulations in Dongguan Children Hospital, Dongguan, CHINA . This project was also approved by HKU, Guangdong Provincial People's Hospital.

Note that full information on the approval of the study protocol must also be provided in the manuscript.

Field-specific reporting

Please select the one below that is the best fit for your research. If you are not sure, read the appropriate sections before making your selection.

☒ Life sciences ☐ Behavioural & social sciences ☐ Ecological, evolutionary & environmental sciences

For a reference copy of the document with all sections, see nature.com/documents/nr-reporting-summary-flat.pdf

Life sciences study design

All studies must disclose on these points even when the disclosure is negative.

Sample size	No statistical methods were chosen to determine sample size. Sample size were chosen to provide sufficient material for experiment and ensure replication of the results with affordable cost.
Data exclusions	No data was excluded from the analysis.
Replication	N/A
Randomization	N/A
Blinding	N/A

Reporting for specific materials, systems and methods

We require information from authors about some types of materials, experimental systems and methods used in many studies. Here, indicate whether each material, system or method listed is relevant to your study. If you are not sure if a list item applies to your research, read the appropriate section before selecting a response.

Materials & experimental systems

n/a	Involved in the study
☒	☐ Antibodies
☐	☒ Eukaryotic cell lines
☒	☐ Palaeontology and archaeology
☒	☐ Animals and other organisms
☒	☐ Clinical data
☒	☐ Dual use research of concern
☒	☐ Plants

Methods

n/a	Involved in the study
☒	☐ ChIP-seq
☐	☒ Flow cytometry
☒	☐ MRI-based neuroimaging

Eukaryotic cell lines

Policy information about cell lines and Sex and Gender in Research

Cell line source(s)	The HEK293T, K562 and mouse NIH/3T3 cell lines are purchased from ATCC. The hESCs line H1 is obtained from WiCell Research Institute (WA01). The B6 murine ESCs were obtained from Prof. Pengtao Liu's lab, HKU.
Authentication	N/A
Mycoplasma contamination	All the cells were tested free-of mycoplasma.
Commonly misidentified lines (See ICLAC register)	N/A

Flow Cytometry

Plots

Confirm that:

☒ The axis labels state the marker and fluorochrome used (e.g. CD4-FITC).

☒ The axis scales are clearly visible. Include numbers along axes only for bottom left plot of group (a 'group' is an analysis of identical markers).

☒ All plots are contour plots with outliers or pseudocolor plots.

☒ A numerical value for number of cells or percentage (with statistics) is provided.

Methodology

Sample preparation	Nuclei were prepared following OmniATAC protocol and resuspended in 0.33x PBS buffer. Next, 76 µL nuclei (~5x10^4) were aliquoted to several 1.5ml Eppendorf DNA LoBind ® Tubes, add 20 µL 5xTAPS-DMF buffer and 4 µL 5 µM indexed Tn5 transposome complex. The tagmentation reactions were performed on a thermomixer at 37°C, 500 rpm for 30 min. Then, 500 µL stop buffer (1xPBS, 1% BSA and 20mM EDTA) were added to quench the reaction on ice for 10 min and transferred to FANS tubes. DAPI was added at a final concentration of 1µg/µL to stain the nuclei before sorting.
Instrument	BD FACS Aria for flow cytometry sorting.
Software	N/A
Cell population abundance	DAPI positive
Gating strategy	No stained nuclei for negative control.

☒ Tick this box to confirm that a figure exemplifying the gating strategy is provided in the Supplementary Information.
